# 2, 3, 4′, 5-tetrahydroxystilbene-2-0-β-d Glycoside Attenuates Age- and Diet-Associated Non-Alcoholic Steatohepatitis and Atherosclerosis in LDL Receptor Knockout Mice and Its Possible Mechanisms

**DOI:** 10.3390/ijms20071617

**Published:** 2019-04-01

**Authors:** Jin Xu, Yi Peng, Yi Zeng, Yi-qiao Hua, Xiao-le Xu

**Affiliations:** Department of Pharmacology, Nantong University Pharmacy College, Nantong, 226001, China; xjntu2603@126.com (J.X.); bennym@163.com (Y.P.); yizeng668@aliyun.com (Y.Z.); hyq971112@163.com (Y.-q.H.)

**Keywords:** nonalcoholic steatohepatitis, atherosclerosis, metabolic syndrome

## Abstract

The compound, 2,3,5,4′-tetrahydroxystilbene-2-O-β-d-glucoside (TSG), a primary bioactive polyphenolic component of *Polygonum multiflorum* exerts numerous pharmacological activities. However, its protective effect against non-alcoholic steatohepatitis (NASH), in the context of metabolic syndrome, remains poorly understood. The aim of the present study is to evaluate the effects of TSG treatment on middle-aged (12-mo-old) male LDLr^−/−^ mice, which were fed a high fat diet for 12 weeks to induce metabolic syndrome and NASH. At the end of the experiment, the blood samples of mice were collected for determination of metabolic parameters. Liver and aorta tissues were collected for analysis, such as histology, immunofluorescence, hepatic lipid content, real-time PCR, and western blot. Our data show that TSG treatment improved the different aspects of NASH (steatosis, inflammation, and fibrosis) and atherosclerosis, as well as some of the metabolic basal characteristics. These modulatory effects of TSG are mediated, at least in part, through regulating key regulators of lipid metabolism (SREBP1c, PPARα and their target genes, ABCG5 and CYP7A1), inflammation (CD68, TNF-α, IL-6 and ICAM), fibrosis (α-SMA and TNFβ) and oxidative stress (NADPH-oxidase 2/4, CYP2E1 and antioxidant enzymes). These results suggest that TSG may be a promising candidate for preventing and treating the progression of NASH.

## 1. Introduction

With the global increase in obesity and metabolic syndrome, the associated complications, such as non-alcoholic steatohepatitis (NASH), atherosclerosis, and diabetes are presenting significant public health burdens in developed countries. NASH is the progressive form of non-alcoholic fatty liver disease (NAFLD) and is defined as hepatosteatosis in concert with inflammation, hepatocyte injury (ballooning), and fibrosis [1]. As one of the most common age-related diseases, NAFLD occurs more often in the middle-aged and elderly. Furthermore, approximately 25% NAFLD patients can progress to NASH and up to a third of these have NASH [2]. NASH is a serious liver disease that can further progress to cirrhosis, hepatocellular cancer, and liver failure and is projected to become the leading cause of liver transplantation in the next several years, particularly since, currently, there are no approved therapeutic strategies for NAFLD/NASH [3].

The pathological progression of NASH has been proposed to follow multiple parallel hits. The first hit involves excessive hepatic steatosis associated with metabolic syndrome, which causes the liver to be vulnerable to any hit that may follow, including inflammation and oxidative stress, thereby leading to liver damage that is associated with increased blood levels of hepatic enzymes, such as aspartate aminotransferase (AST) and alanine aminotransferase (ALT) [4]. The resulting hepatocellular death and necrosis promotes hepatic stellate cell activation, myofibrillar cell infiltration of the liver, and subsequent liver fibrosis. The results from both the pre-clinical and clinical trials distinctly point to a combinatorial approach (lipid regulation, anti-inflammatory, antioxidant, and anti-fibrosis) as a therapeutic remedy for NASH. Nowadays, the absence of approved pharmacological therapies for NASH is a primary incentive for research into developing new potent candidates for this condition [3].

**Figure 1:**
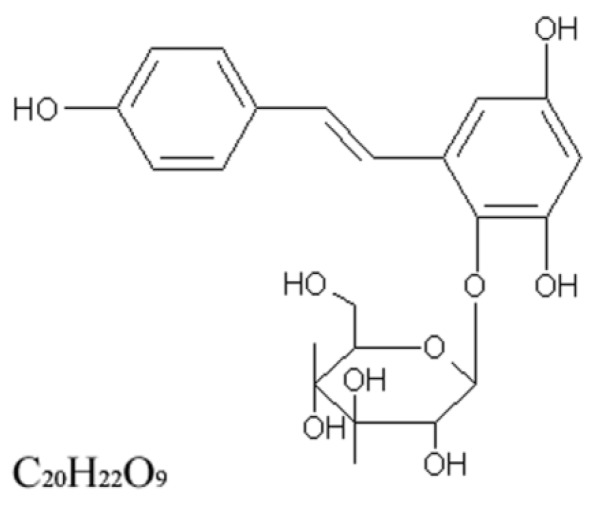
Chemical structure of 2,3,5,4′-tetrahydroxystilbene-2-O-β-D-glucoside (TSG).

The compound, 2,3,5,4′-tetrahydroxystilbene-2-O-β-d-glucoside (TSG, the chemical structure of TSG is shown in Figure 1) is a main bioactive polyphenolic component of *Polygonum multiflorum*, a traditional Chinese medicine widely used as a tonic and anti-aging agent in Asia. Previous studies have demonstrated that TSG, through its favorable therapeutic properties, such as antioxidant and anti-inflammatory properties, are effectively used to treat health related disorders, including cardiac disabilities, diabetes, atherosclerosis, cerebral ischemia, Alzheimer’s disease, and Parkinson’s disease [5,6,7,8,9,10,11,12]. Our previous studies showed that TSG suppressed hepatic steatosis, atherosclerotic lesion formation, and macrophage foam cell formation in ApoE^-/-^ mice [12]. Recently, Ning et al. reported that TSG protected the heart, kidney, and liver in aged mice, consuming excess calories and delayed senile symptoms [13]. Importantly, Han et al. reported that TSG attenuated methionine and choline-deficient diet-induced NAFLD [14]. However, some rare literature reports have looked at whether TSG can attenuate NASH in the model of metabolic syndrome or not. Methionine and choline-deficient-fed mice do not develop metabolic syndrome, instead demonstrating reductions in plasma triglycerides and body weight, and are therefore very different from NASH in human metabolic syndrome or diabetes patients, who are mostly obese and/or hyperlipidemic [15,16]. Conversely, hyperphagic db/db or ob/ob mice develop metabolic syndrome and hepatic steatosis, but not liver fibrosis and inflammation [16,17]. Gupte et al. identified and characterized a novel mouse model, middle-aged male LDLR^-/-^ mice fed high-fat diet (HFD), which developed NASH in the context of metabolic syndrome [16]. These mice develop obesity, dyslipidemia, and diabetes, concurrent with histologic NASH, allowing the investigation of metabolic syndrome-related mechanisms involved in the pathogenesis and treatment of NASH. Thus, the aim of the present study was to investigate the impact of TSG on NASH and its associated metabolic disorders in high-fat diet-fed middle-aged (12-mo-old) LDLR^-/-^ male mice, and to explore the possible underlying mechanisms involved. 

## 2. Results

### 2.1. Effect of TSG on Metabolic Parameter Change

**Figure 2:**
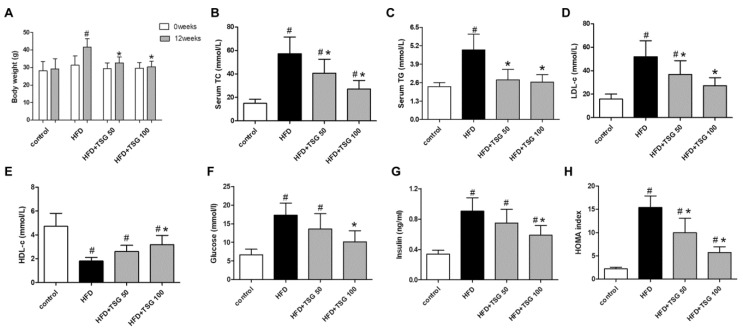
Effect of TSG on metabolic parameter change. (**A**) Body weight for 0 and 12 weeks. (**B**) Serum total cholesterol (TC) concentrations. (**C**) Serum TG concentrations. (**D**) Serum LDL-c concentrations. (**E**) Serum HDL-c concentrations. (**F**) Serum glucose level. (**G**) Serum insulin level. (**H**) Homeostasis model assessment (HOMA) index. *n* = 6. Results are presented as the mean ± S.D. ^#^ *p* < 0.05 vs. control group; * *p* < 0.05 vs. HFD alone group. TSG 50, TSG 50 mg/kg/day; TSG 100, TSG 100 mg/kg/day.

As shown in Figure 2A–E, after 12 weeks of feeding on a HFD, middle-aged male LDLr^−/−^ mice had significantly higher body weight, serum total cholesterol (TC), triglyceride (TG), and low density lipoprotein cholesterol (LDL-c) levels, as well as lower serum high density lipoprotein cholesterol (HDL-c) level compared with standard chow fed mice. Treatment with TSG, both at 50 and 100 mg/kg/day dosages, significantly reduced the body weight, serum TC, TG, and LDL-c levels compared with HFD alone group. The administration with TSG at 100 mg/kg/day dosage also markedly increased the serum HDL-c level, compared with the HFD alone group (Figure 2E). Middle-aged mice demonstrated significant increases in fasting glucose, insulin levels, and homeostasis model assessment (HOMA) index in response to a 12-week HFD (Figure 2F–H). The administration with TSG decreased fasting glucose and insulin levels and HOMA index, especially at 100 mg/kg/day dosage (Figure 2F–H).

### 2.2. Effect of TSG on Hepatic Steatosis

**Figure 3:**
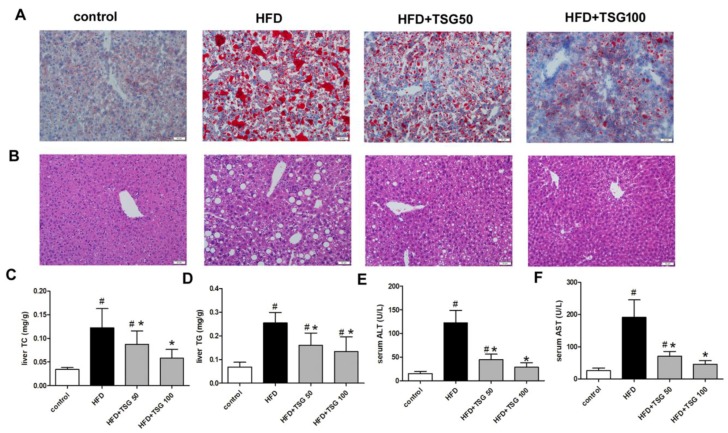
Effect of TSG on hepatic steatosis. (**A**) Representative images of liver sections stained with Oil Red O. Bar = 50 µm. (**B**) Representative images of liver sections stained with H&E. Bar = 50 µm. (**C**) Liver TC content. (**D**) Liver TG content. (**E**) Serum alanine aminotransferase (ALT) level. (**F**) Serum ALT level. *n* = 6. Results are presented as the mean ± S.D. ^#^ *p* < 0.05 vs. control group; * *p* < 0.05 vs. HFD alone group. TSG 50, TSG 50 mg/kg/day; TSG 100, TSG 100 mg/kg/day.

An analysis of fat content on liver sections, stained with Oil Red O, showed that the liver sections of middle-aged LDLr^−/−^ mice fed with HFD, exhibited many macro and micro-lipid droplets, compared with standard chow fed mice (Figure 3A). This finding was further confirmed by H and E staining, thereby demonstrating an increased number and size of intracytoplasmic macro- and micro-vacuoles, within the livers from model groups (Figure 3B). Consistent with the above hepatic histological observations, TC and TG contents in the liver were significantly increased in the model group, compared to the control group (Figure 3C,D). TSG treatment improved the morphology of the liver, and reduced HFD-induced lipid accumulation and hepatic TC and TG levels, compared with HFD-fed alone group (Figure 3A–D). Moreover, treatment with TSG, both at 50 and 100 mg/kg/day dosages, significantly decreased serum ALT and AST levels, compared with HFD-fed alone group (Figure 3E,F). Taken together, these results suggest that TSG reverses hepatic steatosis and reduces liver damage in the HFD middle-aged LDLr^−/−^ mice.

**Figure 4:**
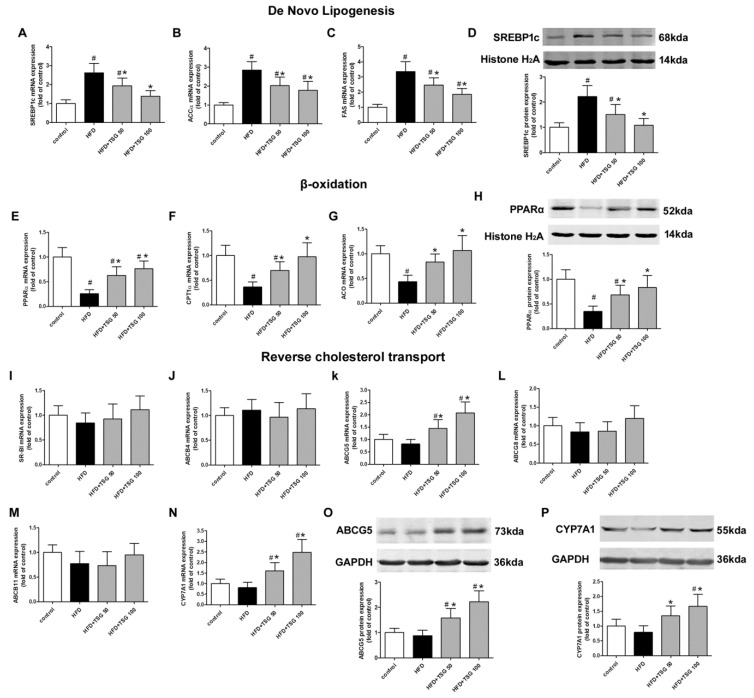
Effect of TSG on protein expression of hepatic genes involved in lipid metabolism. (**A**–**C**) mRNA expression of SREBP1c (**A**), ACCα (**B**) and FAS(**C**) in liver. (**D**) Protein expression of SREBP1c in liver. (E–G) mRNA expression of PPARα (**E**), CPT1α (**F**) and ACO (**G**) in liver. (**H**) Protein expression of PPARα in liver. (I–N) mRNA expression of SR-BI (**I**), ABCB4 (**J**), ABCG5 (**K**), ABCG8 (**L**), ABCB11 (**M**) and CYP7A1 (**N**) in liver. (**O**) Protein expression of ABCG5 in liver. (**P**) Protein expression of CYP7A1 in liver. *n* = 6. Results are presented as the mean ± S.D. ^#^ *p* < 0.05 vs. control group; * *p* < 0.05 vs. HFD alone group. TSG 50, TSG 50 mg/kg/day; TSG 100, TSG 100 mg/kg/day.

To elucidate the underlying mechanism responsible for the inhibitory effect of TSG on hepatic steatosis, we determined expression levels of some key genes involved in *de novo* lipogenesis (sterol regulatory element binding protein1c, SREBP1c; Acetyl-CoA Carboxylase α, ACCα; fatty acid synthase, FAS), lipid β-oxidation (peroxisome proliferator-activated receptor α, PPARα; camitine palmitoyltransferase 1α, CPT1α; Acyl Coenzyme A Oxidase, ACO), reverse cholesterol transport (scavenger receptor class B type I, SR-BI; ATP-binding cassette transporter B4/B11/G5/G8, ABCB4/B11/G5/G8; cholesterol 7alpha-hydroxylase, CYP7A1) and cholesterol biosynthesis (3-hydroxy-3-methylglutaryl-coenzyme A reductase, HMGCR; 3-hydroxy-3-methylglutaryl-coenzyme A synthase, HMGCS). As shown in Figure 4A–D, compared to the model group, treatment with TSG (50 and 100 mg/kg/day) significantly decreased the mRNA and protein levels of SREBP1c. Consistent with the expression pattern of SREBP1c, the mRNA levels of its downstream genes ACCα and FAS significantly decreased in TSG-treated mice group compared to HFD alone group. The mRNA expression levels of CPT1α and ACO, two key enzymes in the lipid β-oxidation, were decreased by the HFD diet and were increased with TSG treatment (Figure 4F,G). Additionally, CPT1α and ACO expression levels are modulated by PPARα. As shown in Figure 4E,H, TSG treatment increased both mRNA and protein expressions of PPARα. Among the hepatic genes involved in reverse cholesterol transport, TSG treatment significantly increased ABCG5 and CYP7A1 mRNA expressions compared to the model group (Figure 4I–N). The alteration of mRNA expressions in ABCG5 and CYP7A1 by TSG treatment was confirmed at protein levels (Figure 4O,P). TSG treatment has little effect on HMGCR and HMGCS gene expressions (Supplementary Figure S1).

### 2.3. Effect of TSG on Hepatic Inflammation

**Figure 5:**
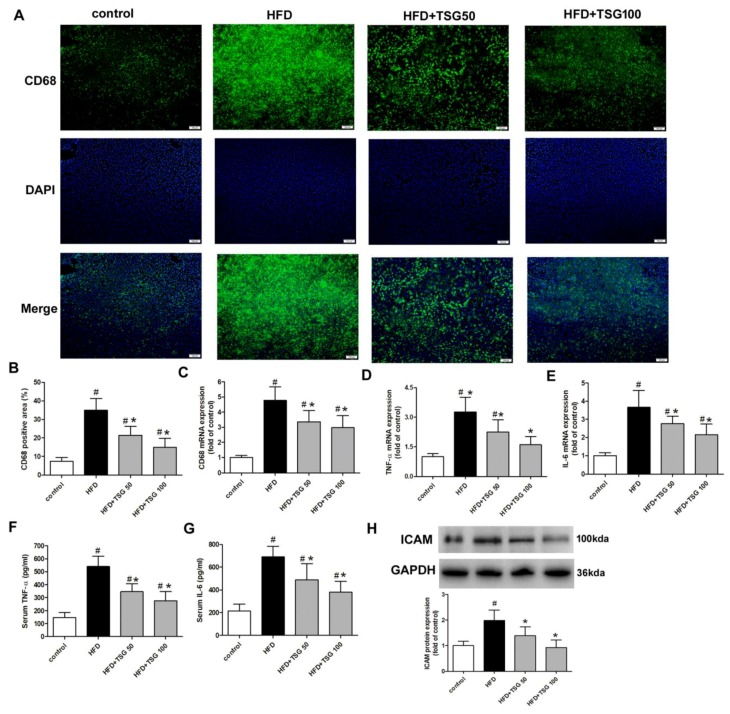
Effect of TSG on hepatic inflammation. (**A**) Representative images of liver sections stained with CD68. Bar = 100 µm. (**B**) Quantification of CD68 positive staining (*n* = 6). (C–E) mRNA expression levels of CD68 (**C**), TNF-α (**D**) and IL-6 (**E**) in liver. (**F** and **G**) Serum levels of TNF-α (**F**) and IL-6 (**G**). (**H**) Protein expression of ICAM in liver. *n* = 6. Results are presented as the mean ± S.D. ^#^ *p* < 0.05 vs. control group; * *p* < 0.05 vs. HFD alone group. TSG 50, TSG 50 mg/kg/day; TSG 100, TSG 100 mg/kg/day.

Macrophages and monocyte populations are an important source of cytokines in the liver and are key players in hepatic inflammation and NASH progression. Middle-aged male LDLr^−/−^ mice, fed with HFD for 12 weeks, indicated significantly increased positive staining area and mRNA expression of CD68 (a marker of macrophage accumulation), compared to the control group (Figure 5A–C). This indicated that there was an increased recruitment of macrophages. Consistent with this finding, mRNA expressions of pro-inflammatory cytokines tumor necrosis factor-α (TNF-α) and interleukin-6 (IL-6) in liver and serum levels of these two pro-inflammatory cytokines were increased in model group (Figure 5D–G). Intercellular adhesion molecule (ICAM) protein expression was also significantly higher at 12 weeks in the HFD-fed alone group compared with the control group (Figure 5H). TSG treatment significantly reversed the increased of the above inflammation-related genes (Figure 5A–H).

### 2.4. Effect of TSG on Hepatic Fibrosis

**Figure 6:**
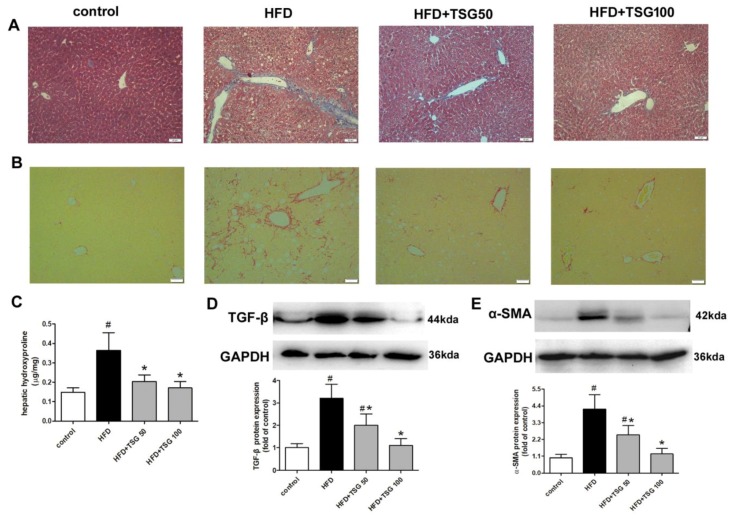
Effect of TSG on hepatic fibrosis. (**A**) Representative images of liver sections stained Masson’s trichrome. (**B**) Representative images of liver sections stained with picro-sirius red. (**C**) Hepatic hydroxyproline content. (D and E) Protein expression of TGFβ (**D**) and α-SMA (**E**) in liver. *n* = 6. Results are presented as the mean ± S.D. ^#^ *p* < 0.05 vs. control group; * *p* < 0.05 vs. HFD alone group. TSG 50, TSG 50 mg/kg/day; TSG 100, TSG 100 mg/kg/day.

One of the features of the advanced stages of NASH is hepatic fibrosis [18]. Middle-aged male LDLr^−/−^ mice, fed with HFD for 12 weeks, exhibited extensive fibrosis, as evidenced by blue collagen staining, using Masson’s trichrome stain, and red collagen staining, using the picro-sirius red stain (Figure 6A,B). Likewise, hepatic hydroxyproline content, a marker of collagen deposition, was significantly increased in the model group compared to the control group (Figure 6C). However, TSG treatment significantly attenuated hepatic fibrosis (Figure 6A–C). The histological findings were confirmed by gene expression analysis. HFD-induced protein expressions of profibrotic and fibrosis-related genes, α-SMA and TGFβ, were markedly attenuated in TSG-treated mice (Figure 6D,E).

### 2.5. Effect of TSG on Hepatic Oxidative Stress

**Figure 7:**
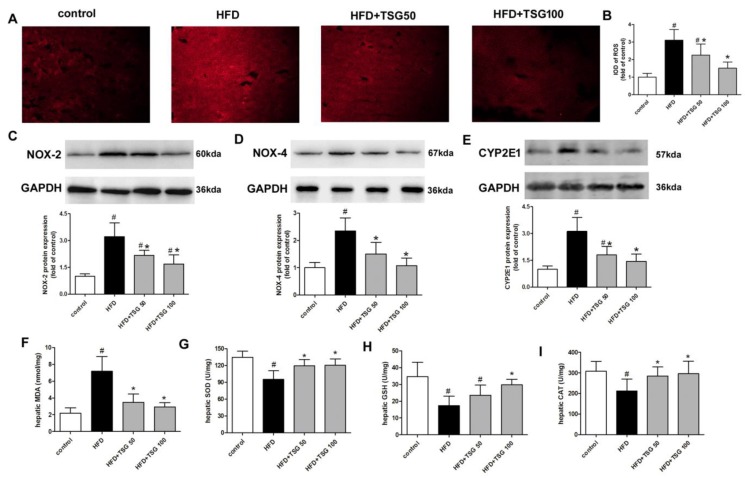
Effect of TSG on hepatic oxidative stress. (**A**) Representative DHE fluorescence staining of liver sections for ROS production (original magnification 100X). (**B**) Quantification of DHE fluorescence image of A. (**C**–**E**) Protein expression of NOX-2, NOX-4 and CYP2E1 in liver. (**F**) MDA content in liver. (**G**) SOD activity in liver. (**H**) GSH activity in liver. (**I**) CAT activity in liver. *n* = 6. Results are presented as the mean ± S.D. ^#^ *p* < 0.05 vs. control group; * *p* < 0.05 vs. HFD alone group. TSG 50, TSG 50 mg/kg/day; TSG 100, TSG 100 mg/kg/day.

Hepatic oxidative stress is a dominant feature of NASH. In situ generation of reactive oxygen species (ROS) was determined by dihydroethidium (DHE) staining within the liver. The result of fluorescence signal indicated that middle-aged male LDLr^−/−^ mice, fed a HFD for 12 weeks, caused higher ROS generation in the liver, while TSG treatment (50 and 100 mg/kg/day) significantly reduced ROS generation (Figure 7A,B). Meanwhile, TSG significantly decreased protein expressions of NADPH oxidase (NOX) subunits NOX-2 and NOX-4 in liver, compared with HFD alone group (Figure 7C,D). Cytochrome p450 2E1 (CYP2E1) is another major source of hepatic ROS [2]. Here, we observed a marked increase in CYP2E1 protein expression in the model group compared with the control group, which was reversed by TSG treatment (Figure 7E). In addition, middle-aged male LDLr^−/−^ mice, which fed a HFD for 12 weeks, developed a higher level of hepatic malondialdehyde (MDA), lower levels of hepatic superoxide dismutase (SOD), and glutathione (GSH) and catalase (CAT) activity, compared to the control group (Figure 7F–I). TSG treatment decreased MDA content and increased the activities of SOD, GSH, and CAT in the liver, compared with the model group, especially at 100 mg/kg/day dosage (Figure 7F–I).

### 2.6. Effect of TSG on Atherosclerotic Lesion Formation

**Figure 8:**
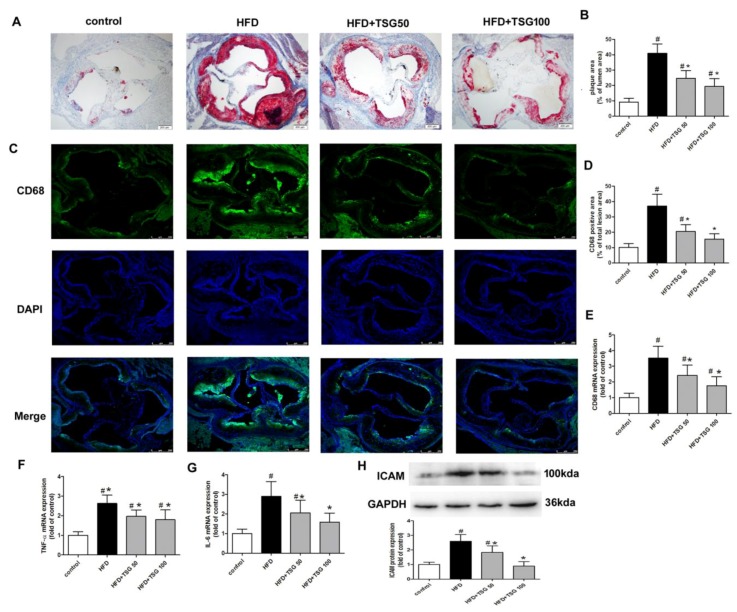
Effect of TSG on atherosclerotic lesion formation. (**A**) and (**B**) representative images and quantification of aortic root sections stained with oil red O. Bar = 200 µm. (**C** and **D**) Representative images of aortic root sections stained with CD68 and quantification of CD68 positive staining. Bar = 250 µm (E–G) mRNA expression levels of CD68 (**E**), TNF-α (**F**) and IL-6 (**G**) in aorta. (**H**) Protein expression of ICAM in aorta. n=6. Results are presented as the mean ± S.D. ^#^ *p* < 0.05 vs. control group; * *p* < 0.05 vs. HFD alone group. TSG 50, TSG 50 mg/kg/day; TSG 100, TSG 100 mg/kg/day.

Atherosclerotic lesion size was determined by quantifying the areas of oil red O staining. The result showed that, TSG treatment obviously inhibited atherosclerotic lesion formation in the aortic root, compared with HFD alone group (Figure 8A,B). Additionally, TSG treatment significantly reduced the intraplaque macrophage infiltration, as indicated by the immunostaining of the macrophage marker CD68, compared with HFD alone group (Figure 8C,D). Middle-aged male LDLr^−/−^ mice fed a HFD developed higher mRNA expressions of CD68, TNF-α and IL-6 and protein expression of ICAM in aorta of mice, while treatment with TSG significantly reversed these increases (Figure 8E–H).

## 3. Discussion

NASH and atherosclerosis share multiple risk factors including, obesity, diabetes, hyperlipidemia, inflammation, and oxidative stress [16]. The LDLR^−/−^ mouse is a widely-used hypercholesterolemic atherosclerosis model. Recently, middle-aged male LDLR^−/−^ mice, fed a high-fat diet, are reported as an ideal model to investigate NASH in the context of metabolic syndrome. These mice develop 4 of 5 criteria of human metabolic syndrome criteria and recapitulate all of the key characteristics of human NASH, including hepatic steatosis, rampant inflammation and fibrosis, underscoring the importance of oxidative stress and age-dependency, which mediate NASH-associated hepatic injury (ALT and AST) [16,19]. This is the first study to evaluate the pharmacological effects of TSG on NASH, and atherosclerosis in HFD-fed middle-aged LDLR^-/-^ mice. In the present investigation, we found that HFD clearly induced a worse metabolic phenotype and substantial acceleration of NASH extent, in middle-aged LDLR^−/−^ mice, while TSG prevented the development of multiple NASH-associated traits, including obesity, lipid imbalance, insulin resistance, hepatic steatosis, inflammation, fibrosis, and elevated liver enzyme levels. In addition, TSG decreased atherosclerotic lesions extent. 

Insulin resistance is associated with various risk factors for NASH, including lipid accumulation, obesity, inflammation, and oxidative stress, which subsequently promote the development of NASH [20]. In the present study, the administration of TSG to HFD-fed middle-aged LDLR^-/-^ mice prevented weight gain, increased fasting glucose and insulin, and HOMA index, suggesting that TSG may ameliorate insulin resistance. Dyslipidemia is frequently observed in patients with atherosclerosis and NASH. Treatment of dyslipidemia plays an important role in the overall management of these patients [21]. This study demonstrated that the administration of TSG increased the level of HDL-c and decreased the serum levels of TG, TC, and LDL-c in HFD-fed LDLr^-/-^ mice. The liver is a central metabolic organ responsible for lipid and lipoprotein metabolism. According to the multiple hit hypothesis, hepatic steatosis is the initial event in the development of NAFLD that can ultimately lead to NASH [4]. The present study showed that the administration of TSG to HFD-fed middle-aged LDLR^-/-^ mice notably reduced the lipid accumulation and steatosis in the liver and prevented liver injury. Next, to further understand the molecular mechanism responsible for the inhibitory effect of TSG on hepatic steatosis, the key molecules involved in lipid metabolism pathways were assessed. De novo lipogenesis is the metabolic pathway that enables the accumulation of fatty acids and triglyceride synthesis in the liver, under condition of a high-fat diet. The levels of de novo lipogenesis are determined by the sterol regulatory element binding protein (Srebp) [22]. SREBP1c is synthesized as an inactive precursor bound to the endoplasmic reticulum membrane. Once activated, SREBP1c is escorted to the Golgi, where SREBP1c is activated to produce the mature form. The active mature form of SREBP enters the nucleus and activates the transcription of its target lipogenic genes [22,23]. Our data showed that TSG treatment significantly decreased the expression of SREBP1c and its downstream lipogenesis genes, ACCα and FAS. Additionally, a decrease in the β-oxidation of fatty acids also leads to HFD-induced accumulation of hepatic lipids. PPARα, an important transcription factor, regulates the expression of CPT1α and ACO, which are two key enzymes in the β-oxidation of fatty acids [24]. TSG treatment upregulated the expressions of PPARα, CPT1α, and ACO. Our results also indicated that TSG markedly up-regulated the expression of hepatic ABCG5 and CYP7A1, which are important factors in reverse cholesterol transport. Reverse cholesterol transport is believed to be an important defense mechanism in hepatic steatosis and atherosclerosis, since it is responsible for the elimination of excess cholesterol from the body [25]. Hepatic ABCG5 mediates the efflux of cholesterol into the bile [26], and CYP7A1 is the primary rate-limiting enzyme of bile acid synthesis in liver [27]. Hence, the regulation of key molecules involved in de novo lipogenesis, lipid β-oxidation, and reverse cholesterol transport by TSG treatment, may contribute to its beneficial effects on serum lipid profiles, hepatic steatosis, and atherosclerosis in HFD-fed middle-aged LDLR^-/-^ mice.

Chronic inflammation is the main pathological difference between hepatic steatosis and NASH. Long-term high-fat diet can recruit Kupffer cells (resident hepatic macrophages) in the liver, which induces the secretion of inflammatory cytokines, such as IL-6 and TNF-α [28]. It has been confirmed that serum and hepatic levels of IL-6 and TNF-α are increased in patients with NASH, and correlate with histological severity of liver damage [4]. Kupffer cells also induce the production of cell adhesion molecules (e.g., ICAM) that recruit monocytes to the liver, further promoting an inflammatory environment in the liver [29]. Reducing inflammation is an obvious target for NASH therapy. In line with the reports about the anti-inflammatory effect of TSG [5,10,12], the results of the present study showed that TSG treatment reduced hepatic inflammatory cell infiltration through the detection of CD68-positive Kupffer cells, and serum and hepatic mRNA levels of IL-6 and TNF-α, as well as ICAM protein expression, which may contribute to its protective effect on NASH. Inflammation is also known to underlie the etiopathogenesis of atherosclerosis. Our data also indicated that TSG treatment inhibited aortic inflammation by decreasing the expression of the above key inflammation-associated genes. 

Liver fibrosis is one of the typical histopathological features in NASH patients, suggesting a more severe and progressive liver injury. As chronic liver injury and inflammatory process, hepatic stellate cells become activated and transdifferentiate to myofibroblast-like cells, leading to deposition of extracellular (fibrotic) matrix [4,18]. Consistent with the fact that mice with metabolic syndrome were used in the present study is an established model that displays all of the hallmarks of NASH, from steatosis to inflammation and fibrosis development [16,19]. The livers from these mice exhibited increased Masson’s trichrome and picro-sirius red staining, as well as collagen deposition indicative of extensive liver fibrosis. The most important finding of our study was the remarkable anti-fibrotic effect, exerted by the TSG in the liver, which has never been reported. Moreover, TSG significantly down-regulated the protein expression of α-SMA, which is a marker of the activated hepatic stellate cells and TGFβ, which is a key pro-fibrosis cytokine in the liver. Our findings agree with previous publications, demonstrating anti-remodeling effects of TSG in cardiac fibrosis [5]. 

Oxidative stress is believed to be a major contributor to the pathogenesis and progression of NASH. Oxidative stress promotes lipid peroxidation and accumulation, stimulates inflammation, and fibrosis through the activation of hepatic stellate cells in the liver, thus accelerating NASH [30]. Oxidative stress is due to an imbalance of oxidants and antioxidants, with an overall increase in cellular levels of ROS. TSG possesses strong antioxidant and free radical-scavenging activities [5]. The present study firstly showed that treatment with TSG significantly reduces the production of endogenous ROS in mice livers. Consistently, the level of MDA, a product of lipid oxidation, decreased in the liver by TSG treatment. Mechanically, NADPH oxidase enzymes are a main source of ROS, especially in that the induction of major isoforms NOX-2 and NOX-4 in hepatocytes leads to apoptosis, further triggering the cascade of events leading to NASH and fibrosis [31]. CYP2E1 is another critically important enzyme linked to oxidative stress in NASH development. It can inhibit fatty acid oxidation in a NADPH-dependent manner, thereby producing pro-oxidant species in NASH. There is also reciprocal regulation between CYP2E1 and NOX [32]. The present study showed that the increased hepatic protein levels of NOX-2, NOX-4, and CYP2E1 were ameliorated by TSG treatment, which may contribute to a decrease in ROS. In addition, TSG also strengthened the endogenous defense against oxidative stress by increasing the activities of antioxidant enzymes SOD, GSH, and CAT in the liver.

The HFD-fed middle-aged LDLR^-/-^ mice, not only developed NASH, but also developed worse atherosclerosis. We further saw attenuation of aortic atherosclerosis in TSG treated mice. We suggest that TSG’s beneficial effects on atherosclerosis was secondary to a favorable systemic lipid profile.

In conclusion, the present study demonstrated for the first time, that TSG treatment improved the different aspects of NASH (steatosis, inflammation and fibrosis) and atherosclerosis, as well as some of the metabolic basal characteristics in a mouse model of metabolic syndrome. These modulatory effects of TSG are mediated, at least in part, by regulating key regulators of lipid metabolism, inflammation, fibrosis, and oxidative stress. These results suggest that TSG may be a promising candidate for preventing and treating the progression of NASH.

## 4. Materials and Methods

A detailed methods section is available in the Supplemental Materials and Methods. All procedures were approved by the animal care and use committee of Nantong University (approval no. NTU-20170301, 1 March 2017) and conformed to the NIH Guide for the Care and Use of Laboratory Animals (NIH publication, 8th edition, 2011). Male LDLr^−/−^ mice on C57BL/6JNju were purchased from Nanjing Biomedical Research Institute of Nanjing University at 5 weeks of age and maintained on a standard chow diet. They were housed under standard laboratory conditions with a 12-h light/12-h dark cycle. Male mice at 12 months of age (middle-aged) were randomly assigned into four groups for 12 weeks: A control group, a high fat diet (HFD) model group, 50 mg/kg/day TSG-treated group, and 100 mg/kg/day TSG-treated group. The dose of TSG used in this study was based on our previous study [12]. The control group was fed a standard chow diet. The HFD model group was given a high fat diet containing 21% fat and 0.21% cholesterol (D12079B, Open Source Diets, Research Diets, Inc., Changzhou, China). The two TSG treatment groups were given the same HFD and dosed daily via intragastric gavage with 50 and 100 mg/kg/day TSG by weight. TSG was suspended in 0.5% carboxymethyl cellulose. Mice, in control and HFD model groups, received the same volume of vehicle gastrically. All groups of mice were sacrificed following 12 weeks of drug delivery. After an overnight fast, the blood samples of mice were collected for determination of serum levels of TC, TG, LDL-c content, HDL-c, insulin, and glucose. Liver and aorta tissues were collected for further analysis, such as histology, immunofluorescence, hepatic lipid content, real-time PCR, and western blot. Most of the methods have been reported in previous studies of our lab [12,33,34,35].

### Statistical Analysis 

The data were represented as mean ± S.D. All values were analyzed by one-way ANOVA, followed by Newman-Keuls multiple comparison test, using Graphpad Prism 5 software (San Giego, CA, USA), and *P* < 0.05 was considered statistically significant. 

## Supplementary Materials

Supplementary materials can be found at https://www.mdpi.com/1422-0067/20/7/1617/s1.

## Abbreviations

NASH	nonalcoholic steatohepatitis
NAFLD	nonalcoholic fatty liver disease
LDLr^−/−^	low density lipoprotein receptor knockout mice
TSG	2,3,5,4′-tetrahydroxystilbene-2-O-β-D-glucoside
SREBP	sterol regulatory element binding protein
FAS	fatty acid synthase
ACCα	Acetyl-CoA Carboxylase α
PPARα	peroxisome proliferator-activated receptor α
CPT1α	camitine palmitoyltransferase 1α
ACO	Acyl Coenzyme A Oxidase
ABCB4/B11/G5/G8	ATP-binding cassette transporter B4/B11/G5/G8
SR-BI	scavenger receptor class B type I
NOX2/4	NADPH oxidase subunit 2/4
CYP2E1	cytochrome p450 2E1
CYP7A1	cholesterol 7alpha-hydroxylase
TC	total cholesterol
TG	triglyceride
LDL-c	low density lipoprotein cholesterol
HDL-c	high density lipoprotein cholesterol
AST	aspartate aminotransferase
ALT	alanine aminotransferase
SOD	superoxide dismutase
GSH	glutathione
CAT	catalase
MDA	malondialdehyde
DHE	Dihydroethidium
H&E	hematoxylin and eosin
TNF-α	tumor necrosis factor-α
IL-6	interleukin-6
