# Supplementary material for: 2, 3, 4′, 5-tetrahydroxystilbene-2-0-β-d Glycoside Attenuates Age- and Diet-Associated Non-Alcoholic Steatohepatitis and Atherosclerosis in LDL Receptor Knockout Mice and Its Possible Mechanisms
Source: Int J Mol Sci. 2019 Apr 1;20(7):1617. doi: 10.3390/ijms20071617 (PMC6479705; doi:10.3390/ijms20071617)
Supplement: Supplementary file 1 [file ijms-20-01617-s001.zip › supplementary files/Supplemental Materials and Methods.docx]

**Supplemental Materials and Methods**

*Drugs*

The TSG used in the present study (C_20_H_22_O_9_; molecular weight 406; purity >98%) was obtained from Nanjing Dliger Medical Technology Co., LTD, China.

*Animal experiment*

All procedures were approved by the animal care and use committee of Nantong University and conformed to the NIH Guide for the Care and Use of Laboratory Animals (NIH publication, 8th edition, 2011). Male LDLr^−/−^ mice on C57BL/6JNju were purchased from Nanjing Biomedical Research Institute of Nanjing University at 5 weeks of age and maintained on a standard chow diet. They were housed under standard laboratory conditions with a 12-hour light/12-hour dark cycle. Male mice at 12 months of age (middle-aged) were randomly assigned into four groups for 12 weeks: a control group, a high fat diet (HFD) model group, 50 mg/kg/day 50 TSG-treated group and 100 mg/kg/day TSG-treated group. The control group was fed a standard chow diet. The HFD model group was given a high fat diet containing 21 % fat and 0.21 % cholesterol (D12079B, Open Source Diets, Research Diets, Inc). The two TSG treatment groups were given the same HFD and dosed daily via intragastric gavage with 50 and 100 mg/kg/day TSG by weight. TSG was suspended in 0.5% carboxymethyl cellulose. Mice in control and HFD model group received the same volume of vehicle gastrically. All groups of mice were sacrificed following 12 weeks of drug delivery. After an overnight fast, the blood samples of mice were collected for determination of serum levels of total cholesterol (TC), triglyceride (TG), low density lipoprotein cholesterol (LDL-c) content, high density lipoprotein cholesterol (HDL-c), insulin and glucose. Liver and aorta tissues were collected for further analysis such as histology, immunofluorescence, hepatic lipid content, real-time PCR and western blot.

*serum lipid proﬁle determination*

At the end of the treatment, mice were sacrificed and blood samples were collected from the inner canthus. After centrifugation for 10minutes at 3000g at 4°C, the serum was obtained and stored at -80°C until use. Serum TC, TG, LDL-c and HDL-c were detected using the biochemical kits (Zhongsheng Bio-tech Co., Ltd, Beijing, China). Fasting insulin was determined using a commercial mouse insulin elisa kit (Millipore, MA). Fasting glucose were measured with a multifunctional biochemistry analyzer Olympus AU2700 (Olympus, Tokyo, Japan). Insulin resistance was evaluated by homeostasis model assessment (HOMA) index as described previously for mice [1]. Serum levels of TNF-α and IL-6 were measured using elisa kits (Shanghai Westang Biotechnology Co. Ltd, Shanghai, China). Serum aspartate aminotransferase (AST) and alanine aminotransferase (ALT) were examined by corresponding assay kits (Nanjing Jiancheng Bioengineering insititue, Nanjing, China).

*Histochemistry*

Liver tissues were fixed in formalin, and paraffin-embedded or frozen sections were prepared at 5 μm thickness. Sections were stained with Oil Red O or haematoxylin and eosin (H&E) according to a standard procedure to detect hepatic steatosis. Sections were stained with Masson’s Trichrome or picro-sirius red according to a standard procedure to detect collagen for fibrosis.

*Determination of hepatic lipid content and hydroxyproline*

For hepatic lipid content measurement, total lipids were extracted from frozen hepatic tissue (approximately 50 mg). Triglyceride and total cholesterol concentrations were determined using the commercial enzyme kits (Zhongsheng Bio-tech Co., Ltd, Beijing, China) and were normalized to liver protein content.

Hepatic hydroxyproline was measured by commercially available kits (Nanjing Jiancheng Bioengineering institute) to quantify liver collagen content.

*Assessment of hepatic oxidative stress*

The level of malondialdehyde and the activities of catalase, glutathione and superoxide dismutase in the liver were measured with enzymatic colorimetric assays using commercial kits (Nanjing Jiancheng Bioengineering Institute, Nanjing, China) according to the manufacturer’s instructions.

Dihydroethidium (DHE, Vigorous Biotechnology Co., Ltd, Beijing, China) was used for in situ detection of ROS in mouse liver and aorta tissue. In brief, fresh cross sections (8 μm) of unfixed but frozen liver and aorta tissue were immediately incubated with 2µmol/L DHE at 37 °C for 30 min in a humidified chamber. Then the red fluorescence intensity excited by green laser was visualized with a fluorescence microscope (Olympus, Tokyo, Japan) and quantiﬁed using Image-ProPlus analysis software.

Quantification of atherosclerosis

Aherosclerotic lesion severity was assessed by serial sections from the aortic root as we previously described [2-4]. In brief, to determine the sinus lesions in aortic root, 8 μm frozen sections of the aortic root with the presence of the aorta valve cups were prepared and stained with Oil Red O solution. Cryosections of each aortic sinus were also used for immunofluorescence staining with antibody against CD68 (Boster, Wuhan, China). The lipid-stained area of total aorta and aortic root sections and CD68 positive staining were all quantiﬁed using Image-Pro software.

*RNA analysis*

mRNA expression of target genes were quantiﬁed by real-time PCR with forward and reverse primers (Supplementary Table 1). Total RNA was extracted using Trizol reagent (Takara, TaKaRa Biotechnology, Dalian, China). Then RNA (1μg) was reverse-transcribed into cDNA using the PrimeScript RT Master Mix Kit (Takara, TaKaRa Biotechnology, Dalian, China). PCRs were performed on the ABI steponeplus real time pcr system (Applied Biosystems, Foster City, CA) using SYBR Green detection chemistry (Takara, TaKaRa Biotechnology, Dalian, China) with the resulting cDNAs. Experimental cycle threshold (Ct) values were normalized to housekeeping gene 18S.

*Western blot analysis*

Western blot analysis was performed as we previously described [2-4]. Equal amounts of protein were separated, transferred onto nitrocellulose membranes (Bio-Rad, USA), immunoblotted with antibodies (Detailed in Supplementary Table 2) and then incubated in the dark with the appropriate IRDye 680RD secondary antibodies (1:15000). The blot was imaged and quantitated using the Odyssey infrared imaging system (LI-COR Biosciences, Inc.). The scanning was started with intensity parameter set to 5 for western blots.

*Statistical analysis*

Data were represented as mean ± S.D. All values were analyzed by one-way ANOVA followed by Newman- Keuls multiple comparison test using Graphpad Prism 5 software, and *P* < 0.05 was considered statistically significant.

**Supplemental Table 1. Primers for Real time qPCR**

| **Gene** |  | **Primer sequence** |
| --- | --- | --- |
| SREBP1c | Forward | 5′- GGAGCCATGGATTGCACATT -3′ |
|  | Reverse | 5′- GGCCCGGGAAGTCACTGT -3′ |
| ACCα | Forward | 5′- GGCCAGTGCTATGCTGAGAT-3′ |
|  | Reverse | 5′- AGGGTCAAGTGCTGCTCCA-3′ |
| FAS | Forward | 5′- TGTGAGTGGTTCAGAGGCAT-3′ |
|  | Reverse | 5′-TTCTGTAGTGCCAGCAAGCT-3′ |
| PPARα | Forward | 5′- CAAGGCCTCAGGGTACCACT-3′ |
|  | Reverse | 5′- TTGCAGCTCCGATCACACTT-3′ |
| CPT1α | Forward | 5′- AGGACCCTGAGGCATCTATT-3′ |
|  | Reverse | 5′-ATGACCTCCTGGCATTCTCC-3′ |
| ACO | Forward | 5′- ATGCCTTTGTTGTCCCTATC -3′ |
|  | Reverse | 5′- CCATCTTCAGGTAGCCATTATC -3′ |
| HMGCR | Forward | 5′- TGGCAGGACGCAACCTCTAT-3′ |
|  | Reverse | 5′- TGACGGCTTCACAAACCACA-3′ |
| HMGCS | Forward | 5′- GCCGTGAACTGGGTCGAA-3′ |
|  | Reverse | 5′- GCATATATAGCAATGTCTCCTGCAA-3′ |
| SR-BI | Forward | 5′- ATCTGGTGGACAAATGGAA-3′ |
|  | Reverse | 5′- GAAGCGATACGTGGGAAT-3′ |
| ABCG5 | Forward | 5′- AGCGTCAGCAACCGTGTC-3′ |
|  | Reverse | 5′- AGCAGCGTGGTCTTCCCT-3′ |
| ABCG8 | Forward | 5′- TTAAGCCACTCCCAATACA-3′ |
|  | Reverse | 5′- GTTGCTCCAAGAATAAATGA-3′ |
| ABCB4 | Forward | 5′- CCCCACAGAGGGTAAGAT-3′ |
|  | Reverse | 5′- CCAACCAGGGTGTCAAAT-3′ |
| ABCB11 | Forward | 5′- CAAATAAGGTTGTGGGTAA-3′ |
|  | Reverse | 5′- AGGACTGACAGCGAGAAT-3′ |
| CYP7A1 | Forward | 5′-AGCAACTA AACAACCTGCCAGTACTA-3′ |
|  | Reverse | 5′- GTCCGGATATTCAAGGATGCA-3′ |
| CD68 | Forward | 5′-ATCCCCACCTGTCTCTCTCA-3′ |
|  | Reverse | 5′-TTGCATTTCCACAGCAGAAG-3′ |
| TNF-α | Forward | 5′-CGTCAGCCGATTTGCTATCT-3′ |
|  | Reverse | 5′-CGGACTCCGCAAAGTCTAAG-3′ |
| IL-6 | Forward | 5′-AGTTGCCTTCTTGGGACTGA-3′ |
|  | Reverse | 5′-TCCACGATTTCCCAGAGAAC-3′ |
| 18s | Forward | 5′-CGCGGTTCTATTTTGTTGGT-3′ |
|  | Reverse | 5′-AGTCGGCATCGTTTATGGTC-3′ |

**Supplemental Table 2**

Antibodies for Western blotting

| **Antibody** | **Company** | **Country** | **Cat. No.** |  |
| --- | --- | --- | --- | --- |
| NOX-2 | Abcam. | USA | ab129068 |  |
| NOX-4 | abcam | USA | ab133303 |  |
| SREBP1 | abcam | USA | ab28481 |  |
| PPARα | Abcam | USA | ab8934 |  |
| CYP7A1 | Abcam | USA | ab65596 |  |
| CYP2E1 | ProteinTech | China | 19937-1-AP |  |
| FASN | ProteinTech | China | 10624-2-AP |  |
| ABCG5 | ProteinTech | China | 27722-1-AP |  |
| TGF-β | ProteinTech | China | 21898-1-AP |  |
| α-SMA | ProteinTech | China | 55135-1-AP |  |
| ICAM-1 | ProteinTech | China | 60299-1-Ig |  |
| Histone H_2_A | Cell signaling | USA | 3636 |  |
| GAPDH | Kangchen | China | kc5G4 |  |

**Legends for supplemental figure 1**

Effects of TSG on mRNA expression of HMGCR and HMGCS in the liver. n=6. Results are presented as the mean ± S.D. ^#^*P* < 0.05 vs. control group. TSG 50, TSG 50mg/kg/day; TSG100, TSG 100mg/kg/day.

**References**

1. [Mulvihill, E.E](https://www.ncbi.nlm.nih.gov/pubmed/?term=Mulvihill%20EE%5BAuthor%5D&cauthor=true&cauthor_uid=19592617).; [Allister, E.M](https://www.ncbi.nlm.nih.gov/pubmed/?term=Allister%20EM%5BAuthor%5D&cauthor=true&cauthor_uid=19592617).; [Sutherland, B.G](https://www.ncbi.nlm.nih.gov/pubmed/?term=Sutherland%20BG%5BAuthor%5D&cauthor=true&cauthor_uid=19592617).; [Telford, D.E](https://www.ncbi.nlm.nih.gov/pubmed/?term=Telford%20DE%5BAuthor%5D&cauthor=true&cauthor_uid=19592617).; [Sawyez, C.G](https://www.ncbi.nlm.nih.gov/pubmed/?term=Sawyez%20CG%5BAuthor%5D&cauthor=true&cauthor_uid=19592617).; [Edwards, J.Y](https://www.ncbi.nlm.nih.gov/pubmed/?term=Edwards%20JY%5BAuthor%5D&cauthor=true&cauthor_uid=19592617).; [Markle, J.M](https://www.ncbi.nlm.nih.gov/pubmed/?term=Markle%20JM%5BAuthor%5D&cauthor=true&cauthor_uid=19592617).; [Hegele, R.A](https://www.ncbi.nlm.nih.gov/pubmed/?term=Hegele%20RA%5BAuthor%5D&cauthor=true&cauthor_uid=19592617).; [Huff, M.W](https://www.ncbi.nlm.nih.gov/pubmed/?term=Huff%20MW%5BAuthor%5D&cauthor=true&cauthor_uid=19592617). Naringenin prevents dyslipidemia, apolipoprotein B overproduction, and hyperinsulinemia inLDL receptor-null mice with diet-induced insulin resistance. [*Diabetes*.](https://www.ncbi.nlm.nih.gov/pubmed/?term=Naringenin+Prevents+Dyslipidemia%2C+Apolipoprotein+B+Overproduction%2C+and+Hyperinsulinemia+in+LDL+Receptor%E2%80%93Null+Mice+With+Diet-Induced+Insulin+Resistance) 2009, *58*, 2198-210.
2. [Chen, X](https://www.ncbi.nlm.nih.gov/pubmed/?term=Chen%20X%5BAuthor%5D&cauthor=true&cauthor_uid=28863273).; [Tang, K](https://www.ncbi.nlm.nih.gov/pubmed/?term=Tang%20K%5BAuthor%5D&cauthor=true&cauthor_uid=28863273).; [Peng, Y](https://www.ncbi.nlm.nih.gov/pubmed/?term=Peng%20Y%5BAuthor%5D&cauthor=true&cauthor_uid=28863273).; [Xu, X](https://www.ncbi.nlm.nih.gov/pubmed/?term=Xu%20X%5BAuthor%5D&cauthor=true&cauthor_uid=28863273). 2,3,4',5-tetrahydroxystilbene-2-O-β-d-glycoside attenuates atherosclerosis in apolipoprotein E-deficient mice: role of reverse cholesterol transport. [*Can. J. Physiol. Pharmacol.*](https://www.ncbi.nlm.nih.gov/pubmed/?term=2%2C3%2C4%27%2C5-tetrahydroxystilbene-2-O-%CE%B2-d-glycoside+attenuates+atherosclerosis+in+apolipoprotein%E2%80%89E-deficient+mice%3A+role+of+reverse+cholesterol+transport) 2018, *96*, 8-17.
3. Liu, T.T.; Zeng, Y.; Tang, K.; Chen, X.; Zhang, W.; Xu, X.L.. Dihydromyricetin ameliorates atherosclerosis in LDL receptor deficient mice. *Atherosclerosis.* 2017, *262*, 39-50.
4. Tang, K.; Wang, F.; Zeng, Y.; Chen, X.; Xu, X. Salusin-α attenuates hepatic steatosis and atherosclerosis in high fat diet-fed low densitylipoprotein receptor deficient mice. *Eur. J. Pharmacol.* 2018, *830*, 76-86.
