# Supplementary figures and images for: 2, 3, 4′, 5-tetrahydroxystilbene-2-0-β-d Glycoside Attenuates Age- and Diet-Associated Non-Alcoholic Steatohepatitis and Atherosclerosis in LDL Receptor Knockout Mice and Its Possible Mechanisms
Source: Int J Mol Sci. 2019 Apr 1;20(7):1617. doi: 10.3390/ijms20071617 (PMC6479705; doi:10.3390/ijms20071617)

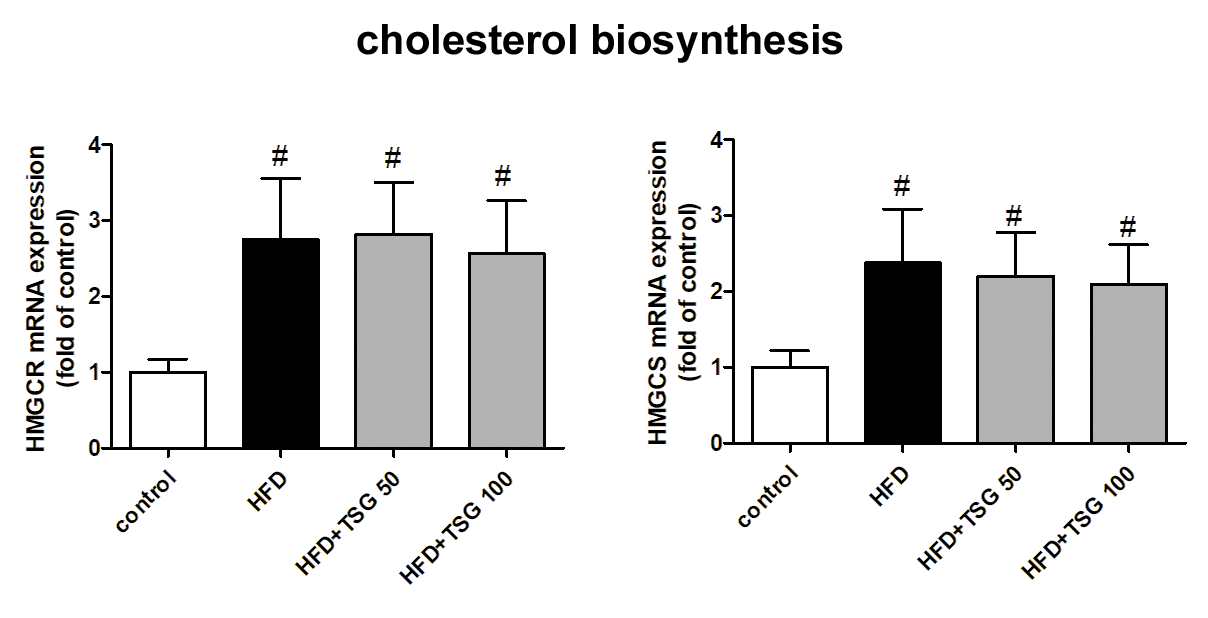

Supplement: Supplementary file 1 [file ijms-20-01617-s001.zip › supplementary files/Supplemental Figure 1.tif]
